# Combination of simple advancement flap and fistulectomy to treat complex anal fistula as a complication of hemorrhoidectomy: Case report

**DOI:** 10.1016/j.amsu.2021.103203

**Published:** 2021-12-21

**Authors:** Imam Sofii, Irianiwati, Gunadi, Adeodatus Yuda Handaya, Aditya Rifqi Fauzi

**Affiliations:** a Digestive Surgery Division, Department of Surgery, Faculty of Medicine, Universitas Gadjah Mada/Dr. Sardjito Hospital, Yogyakarta, 55281, Indonesia; b Department of Anatomical Pathology, Faculty of Medicine, Universitas Gadjah Mada/Dr. Sardjito Hospital, Yogyakarta, 55281, Indonesia; c Pediatric Surgery Division, Department of Surgery, Faculty of Medicine, Universitas Gadjah Mada/Dr. Sardjito Hospital, Yogyakarta, 55281, Indonesia

**Keywords:** Anal stenosis, Post-hemorrhoidectomy, Anal fistula, Advancement flap, Severe

## Abstract

**Introduction:**

After hemorrhoidectomy, anal stenosis occurs, which is an uncommon but severe consequence. The majority of severe cases require advancement flap anoplasty.

**Presentation of case:**

A 50-year-old female patient with a history of hemorrhoidectomy 10 months prior to admission complained of difficulty defecating, pain, and incomplete evacuation sensation, as well as a hole on the right side of the anal canal through which feces unintentionally passed. On the physical examination, we found that the anal lumen was partially obstructed, which did not allow the insertion of a finger. There was an impression of a perineal fistula at 5 and 7 o'clock, which was connected to the anal canal 3 cm from the edge of the anus. The patient was diagnosed with severe anal stenosis with perianal fistula. The patient underwent fistulectomy and advancement flap with perianal skin. In the outpatient follow-up clinic in the first and second weeks, the patient showed no complications, and no recurrence of her complaints was found.

**Discussion:**

Several corrective surgical techniques have been applied to restore a healthy lining to the constricted portion of the anal canal. We performed a combination of simple cutaneous advancement flap and fistulectomy to manage the patient with severe anal stenosis following hemorrhoidectomy with concurrent anal fistula.

**Conclusion:**

A combination of fistulectomy and simple cutaneous advancement flap anoplasty is a simple, safe, and effective surgical option for the management of severe anal stenosis with concomitant anal fistula.

## Highlights

• Uncommon but severe consequence of hemorrhoidectomy is anal stenosis.
• A combination of fistulectomy and simple cutaneous advancement flap anoplasty is a simple, safe, and effective.
• First to report the combination of surgical technique in managing severe anal stenosis.

## 1 Introduction

Anal stenosis is an uncommon but serious anal surgical complication. It usually occurs following a hemorrhoidectomy [1,2]. The constriction of the anal canal caused by various degrees of epithelial lining change into fibrous connective tissue is the hallmark of this rare disorder. Anal stenosis can occur as a result of any disease that produces scarring in the anoderm, but it is most commonly linked to surgical trauma [3,4]. It might be mild, moderate, or severe depending on the degree of stenosis. Surgical anoplasty is required for the majority of severe stenosis cases. Anal stenosis is routinely treated with surgery employing a variety of flaps, including the Y–V, V–Y, lateral, diamond-shaped, and house-type flaps [5]. However, so far, there have been no studies discussing simple cutaneous advancement flaps in severe anal stenosis. Here, we present a case of a patient who developed severe anal stenosis after hemorrhoidectomy and underwent surgery using simple cutaneous advancement flaps. This report has been presented in line with the SCARE criteria [6].

## 2 Presentation of case

**Fig. 1:**
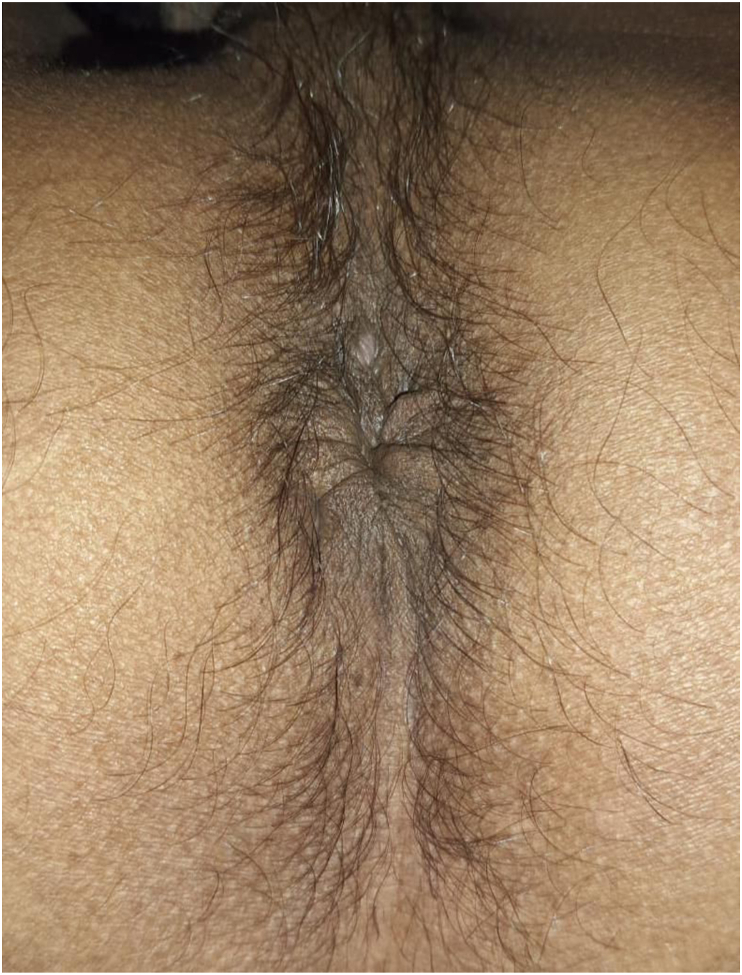
Pre-operative clinical picture.

**Fig. 2:**
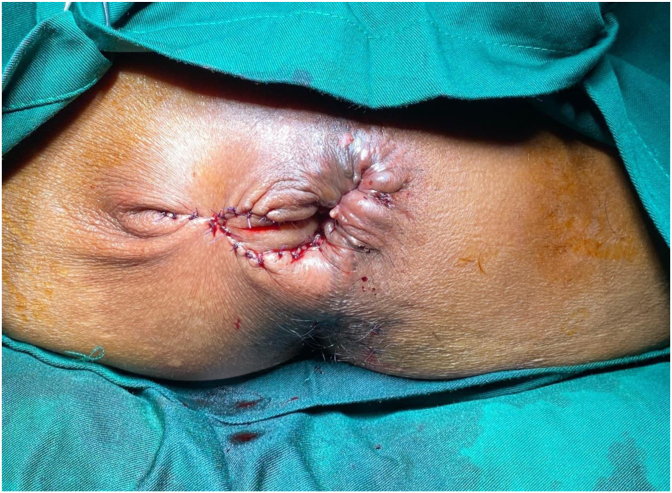
Intra-operative clinical picture.

A 50-year-old female patient with a history of hemorrhoidectomy 10 months before admission had complaints of difficulty in defecating accompanied by pain and incomplete evacuation sensation. The complaint was accompanied by a hole on the right of the anal canal through which feces unintentionally passed. From physical examination, it was found that the anal lumen was partially obstructed, which did not allow the insertion of a finger (Fig. 1), and there was an impression of a perineal fistula at 7 o'clock (5 mm diameter), and 5 o'clock (5 mm diameter) which was connected to the anal canal 3 cm from the edge of the anus. No follow-up examinations were conducted. The diagnosis was confirmed as severe anal stenosis with perianal fistula. The patient underwent fistulectomy and an advancement flap with perianal skin (Fig. 2). The operation was performed one step at a time to repair the fistula and flap with prophylactic antibiotics, intravenous cefazolin 1 g and intravenous metronidazole 500 mg. Exploration of the operating field was done with the Lone Star Retractor™ (The Woodlands, Texas, US). A fistulectomy was performed until the healthy tissue border. Next, a flap is made with a design as shown in the picture. Fixation of the flap in the anal canal was done using absorbable sutures and fixation with the skin using non-absorbable sutures. Analogous to postoperative patency, anal canal diameter was re-examined to ensure postoperative patency. The surgical wound was covered with thick gauze with sufficient pressure to prevent hematoma, infection, and accelerate wound healing. The patient was treated for 1 day to ensure that the patient was physically and psychologically ready for self-care.

**Fig. 3:**
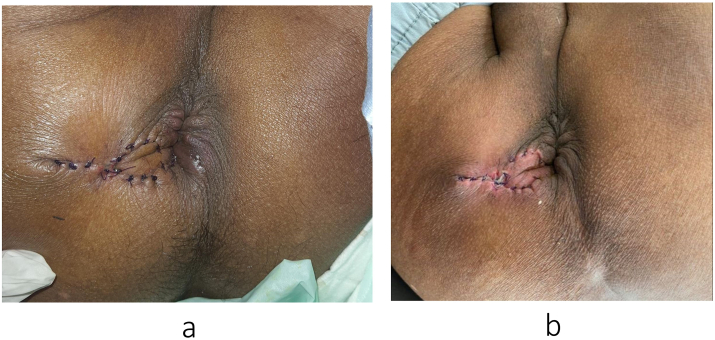
a) 1-week post-operative follow-up; b) 2 weeks post-operative follow-up.

At the postoperative outpatient follow-up in the first (Fig. 3a) and second (Fig. 3b) weeks, the patient showed no complications, and no recurrence of her complaints was found.

## 3 Discussion

Anal stenosis is a rare complication in patients with a history of hemorrhoidectomy. This condition causes discomfort to the patient and is considered a serious complication [7]. In one long-term study, it was found that >10 years after hemorrhoidectomy, patients complained of impaired defecation and a sensation of anal constriction [8].

To restore a healthy lining to the constricted portion of the anal canal, several corrective surgical techniques have been used [9,10]. Complex procedures such as S-plasty are no longer widely used because of significant morbidity and lengthy hospital stays. However, similar approaches may be utilized to treat severe high strictures associated with mucosal ectropion when other treatments have failed, or when a large amount of skin must be excised and new skin rotated into the area, as in the treatment of Paget's disease [10,11].

In this case, a fistula was found at 5 o'clock. This situation became a consideration for making a flap design that could simultaneously lift the fistula channel in stage 1 of surgery. This technique has several risks, such as infection after surgery, more difficult treatment after surgery, and the most severe is flap failure. In addition, the possibility of postoperative infection is very high, considering that there is a fistula which is usually an area of serious infection. Therefore, prophylaxis with a 1st generation cephalosporin broad-spectrum antibiotic such as cefazolin 1g combined with an antibiotic for anaerobic bacteria is administered 30 minutes before incision [12].

The requirements to make a good flap include good vascularization of the pedicle, less tension, free of infection and hematoma, appropriate flap thickness, good post-flap care and supervision, and not interfering with vascularization and neurologic function after the flap [13]. The perianal area has the advantages of high vascularity, more flexible loose connective tissue, and a relatively thick layer of skin along with a thick layer of fatty tissue. This situation is very advantageous in making flaps in the perianal region. Fixation of the flap in the anal canal was done using long absorbable sutures and fixation with the skin using non-absorbable sutures [13]. Canal caliber and diameter were evaluated to ensure postoperative patency. The surgical wound was covered with thick gauze with sufficient pressure to prevent hematoma, infection, and accelerate wound healing [13].

Each of the surgical methods is safe to employ and has been utilized with varying success rates. Because prospective studies have not been undertaken, it is highly difficult to evaluate the findings of numerous anaplastic techniques in the literature [5]. There was no controlled research on the benefits and drawbacks of different anaplastic techniques; nonetheless, practically any treatment will at the very least relieve the patient's symptoms [5]. C anoplasty was utilized by Oh and Zinberg [14] in 12 patients with anal stenosis (10 by prior hemorrhoidectomy, 1 by fistulectomy, and 1 by fissurectomy), and 11 patients had good outcomes, with a total healing rate of 91%. In a research published by Khubchandani [15], 53 patients received mucosal advancement flap anoplasty, with a 94% recovery rate. In two studies [16,17], similar outcomes were found in a total of 33 patients treated with Y–V anoplasty. In a total of 23 patients with anal stricture and mucosal ectropion, diamond flap anoplasty resulted in a 100% healing rate. In 53 patients with anal stenosis and ectropion who had island flap anoplasty, the healing rate was 91.5% [18,19].

Between 1991 and 1995, Aitola [20] did a retrospective analysis on ten patients who received Y–V anoplasty coupled with internal sphincterotomy. All but one patient improved during a 12-month follow-up period. Six patients had good outcomes, three had medium outcomes, and one had a bad outcome. Later on, this patient developed a restenosis. The overall healing rate was 60%, with a 30% improvement rate. A Y–V anoplasty was performed in 29 patients and a diamond flap anoplasty in the remaining 13 cases in a recent study [21]. At a 2-year follow-up, all patients who received diamond flap anoplasty had their stenosis completely resolved (healing rate 100%). 26 (90%) of the 29 patients who underwent Y–V anoplasty thought their clinical outcomes were excellent, whereas three (10%) needed to utilize anal dilators on a regular basis.

In a research published by Rakhmanine et al. [22], 95 patients received lateral mucosal advancement anoplasty. The average time between follow-ups was 50 months. Only 63% of patients had had previous surgery: 35 had had a hemorrhoidectomy, 10 had an anal fissure operation, 4 had a fistula operation, 1 had a transversal excision of a tumor, and 10 had other surgeries. Complications were reported at a rate of 3% overall (one abscess and two seepage of liquid stool).

Previous research on the use of a simple advancement flap technique to treat chronic anal fissures has yielded positive outcomes [23]. To the best of our knowledge, this report is the first to report the combination of simple cutaneous advancement flap with fistulectomy in managing severe anal stenosis following hemorrhoidectomy with concomitant anal fistula.

## 4 Conclusions

A combination of fistulectomy and simple cutaneous advancement flap anoplasty is a simple, safe, and effective surgical option for the management of severe anal stenosis with concomitant anal fistula. However, further research with a larger sample is needed to confirm and clarify our methods.

## Ethical approval

The informed consent form was declared that patient data or samples will be used for educational or research purposes. Our institutional review board also do not provide an ethical approval in the form of case report.

## Sources of funding

The authors declare that this study had no funding source.

## Author contribution

Imam Sofii conceived the study and approved the final draft. Aditya Rifqi Fauzi drafted the manuscript. Irianiwati, Gunadu, and Adeodatus Yuda Handaya critically revised the manuscript for important intellectual content. All authors read and approved the final draft. All authors facilitated all project-related tasks.

## Registration of research studies

The manuscript is a case report, not considered a formal research involving participants.

## Consent

Written informed consent was obtained from the patient for publication of this case report and accompanying images. A copy of the written consent is available for review by the Editor-in-Chief of this journal on request.

## Guarantor

Imam Sofii.

## Provenance and peer review

Not commissioned, externally peer-reviewed.

## Declaration of competing interest

No potential conflict of interest relevant to this article was reported.
